# Identification and field verification of an aggregation pheromone from the white-spotted flower chafer, *Protaetia brevitarsis* Lewis (Coleoptera: Scarabaeidae)

**DOI:** 10.1038/s41598-021-01887-y

**Published:** 2021-11-16

**Authors:** Xiaofang Zhang, Liuyang Wang, Chunqin Liu, Yongqiang Liu, Xiangdong Mei, Zhongyue Wang, Tao Zhang

**Affiliations:** 1 Institute of Plant Protection, Hebei Academy of Agriculture and Forestry Sciences, Integrated Pest Management Center of Hebei Province, Key Laboratory of IPM on Crops in Northern Region of North China, Ministry of Agriculture and Rural Affairs, Baoding, 071000 People’s Republic of China; 2 State Key Laboratory for Biology of Plant Diseases and Insect Pests, Institute of Plant Protection, Chinese Academy of Agricultural Sciences, Beijing, 100193 People’s Republic of China; 3 Cangzhou Technical College, Cangzhou, 061001 People’s Republic of China

**Keywords:** Animal behaviour, Entomology

## Abstract

The white-spotted flower chafer (WSFC), *Protaetia brevit*arsis Lewis, is native to East Asia. Although their larvae are considered a potential resource insect for degrading plant residues, producing protein fodder, and processing to traditional medicine, adult WSFCs inflict damage to dozens of fruit and economic crops. The control of the WSFC still relies heavily on pesticides and the inefficient manual extraction of adults. Here, we report the identification and evaluation of the aggregation pheromone of WSFCs. From the headspace volatiles emitted from WSFC adults, anisole, 4-methylanisole, 2-heptanone and 2-nonanone were identified as WSFC-specific components. However, only anisole and 4-methylanisole elicited positive dose–response relationship in electroantennography tests, and only 4-methylanisole significantly attracted WSFCs of both sexes in olfactometer bioassays and field experiments. These results concluded that 4-methylanisole is the aggregation pheromone of WSFCs. Furthermore, we developed polyethylene vials as long-term dispensers of 4-methylanisole to attract and kill WSFCs. The polyethylene vial lures could effectively attracted WSFCs for more than four weeks. Pheromone-based lures can be developed as an environmentally friendly protocol for monitoring and controlling WSFC adults.

## Introduction

The white-spotted flower chafer (WSFC), *Protaetia brevitarsis* Lewis (Coleoptera: Scarabaeidae: Cetoniinae) (NCBI: txid348688) is native to East-Asia and distributed throughout Japan, Korea, Mongolia and China^1^. Since its larvae usually feed on humus and decomposed plant tissues in topsoil^2^, WSFCs are cultivated as a potential resource insect to produce high-quality organic fertilizer by converting herbaceous and ligneous plant residues^3,4^. In China and Korea, WSFC larvae are also utilized to produce protein fodder and traditional medicine^5–7^. However, WSFC adults, which cause up to 5–30% yield loss to corn, grape and sunflower in the absence of control measures, are considered important agricultural and horticultural pests^8,9^. Although there have been outbreaks of the WSFCs that have caused damage to economic crops in China during the last three decades, there are currently no reliable control strategies for use against WSFCs. Commonly, WSFC larvae spend most of their lives underground often distant from orchards^3^, while adults are excellent flyers that have the ability to colonize feeding and breeding sites rapidly^10^. Another issue that weakens the control efficacy is that the adults preferentially attack ripe or nearly ripe fruits that cannot safely be sprayed with insecticides^10^. To date, control of WSFCs still relies heavily on environmentally damaging pesticides and inefficient manual extraction of adults, which is impossible for large-scale production. Therefore, novel and effective strategies for controlling and monitoring WSFCs are urgently needed.

Trapping and killing of insect pests based on chemical communication cues, such as insect pheromones and food attractants, has been demonstrated as an environmentally friendly method to control agricultural pests^11^. A previous study on the chemical communication of Scarabaeoidea beetles identified aggregation and sex pheromones of more than 50 species in four subfamilies (Dynastinae, Rutelinae, Melolonthinae and Cetoniinae)^12,13^. Unfortunately, pheromones of only a few species of Cetoniinae, have been reported, including a contact pheromone (*Z*)-9-pentacosene in the large hive beetle *Oplostomus haroldi* Witte^14^, (*R*)-γ-decalactone (a male-produced sex pheromone) in an endangered saproxylic beetle *Osmoderma eremita* Scopoli^15^, and phenylacetaldehyde (a female-produced sex pheromone) in the sorghum chafer *Pachnoda interrupta* Olivier^16^.

Considering that WSFC adults are the main threat to economic crops, attracting and killing adults should be an effective method for their control. Several studies have attempted to develop WSFC attractants, mainly based on food volatiles. Historically, mixtures of liquor, sugar and vinegar have been applied as WSFC attractants^8,17^, but their efficiency was unsatisfactory. Japonilure, the sex pheromone of the Japanese beetle, *Popillia japonica*, has also been reported to display attractive qualities^18^. To protect corn in northern China, Chen and Li^19^ developed a novel WSFC control scheme that combined plant volatile attractants and pesticides. However, the chemical communication system of the WSFCs remains unclear. Although it is well known that WSFCs use pheromones for intraspecific communication^20^, no sex pheromone or aggregation pheromone has been identified and reported thus far.

In the present study, we report the existence of an aggregation pheromone in WSFCs, the identification of candidate components of aggregation pheromones, and the evaluation of pheromones in the field. The results could provide valuable data for developing a novel and efficient method to monitor and control WSFCs.

## Results

### Traps baited with live beetles

**Figure 1:**
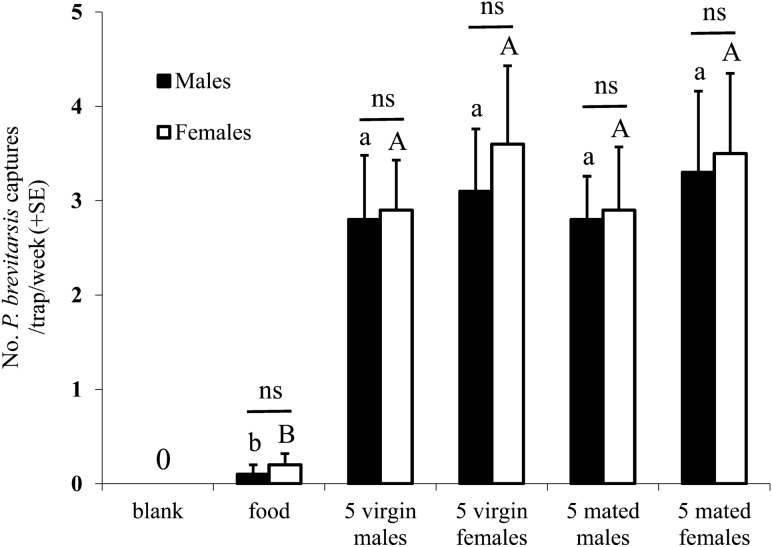
Captures of *Protaetia brevitarsis* in bucket traps baited with virgin males and females (26 July to 9 August 2016). Bars with the same letter present no significant difference (*P* > 0.05).

Both male and female WSFCs were attracted to the bucket traps baited with live beetles, and the captures of WSFC males and females were approximately equal in all treatments (Fig. 1), suggesting that aggregation pheromones rather than sex pheromones, existed in the WSFC population. Neither female nor male WSFCs were caught in the blank traps during any of the field experiments. In the field experiment of verifying the existence of long-distance pheromones, no deaths or escapes of the marked beetles were recorded over a one-day interval, indicating that the captured WSFCs had no opportunities to escape from the bucket traps. Among five traps for two weeks, only two females and two males were captured in the traps baited with food alone. The captures among traps baited with virgin males, virgin females, mated males and mated females were not significantly different (male captures: *F*_*3,16*_ = 0.129, *P* = 0.941; female captures: *F*_*3,16*_ = 0.265, *P* = 0.850) (Fig. 1), indicating that both male and female WSFCs produce and release the aggregation pheromone. It was also concluded that both virgin and mated beetles attracted similar numbers of WSFCs (Fig. 1).

### Volatile identification

**Figure 2:**
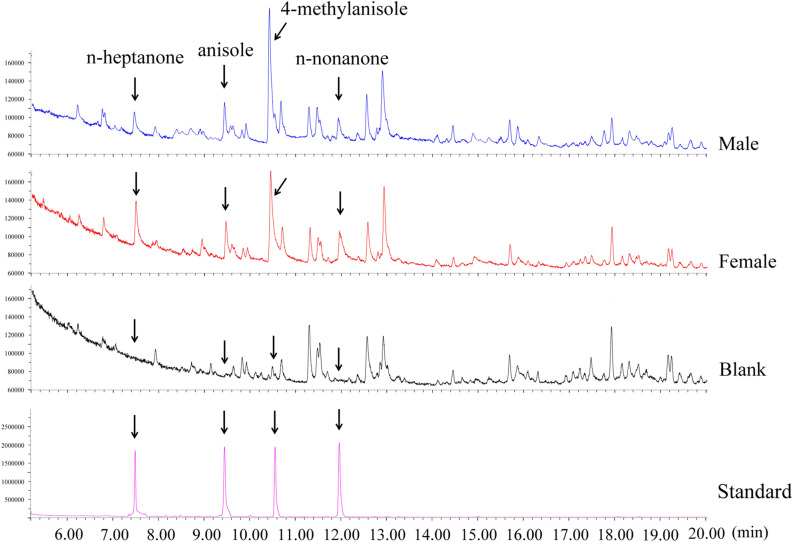
Total ion chromatograms of the volatiles collected from *Protaetia brevitarsis* males (blue), females (red), blank chamber (black), and authentic standards of n-heptanone, anisole, 4-methylanisole and n-nonanone (purple).

Considering that WSFCs are diurnal animals, we collected the volatiles during the photoperiod. The GC–MS analysis revealed that the volatiles from both female and male WSFCs were not large in amounts, their total ion chromatogram (TIC) peaks were easily confused by noisy signals (including column bleeding caused by moisture from collection surroundings, and impurity compounds from environment and solvent). To distinguish the pheromones from beetles, we compared the volatiles of WSFCs with those of blank control (Fig. 2). The comparison results showed that four compounds were identified from the volatile extraction from both male and female WSFCs, including one major component (4-methylanisole) and three minor components (anisole, n-heptanone and n-nonanone). All compounds were verified with authentic samples through retention time and mass spectrometry.

### Electroantennography (EAG) analysis

**Figure 3:**
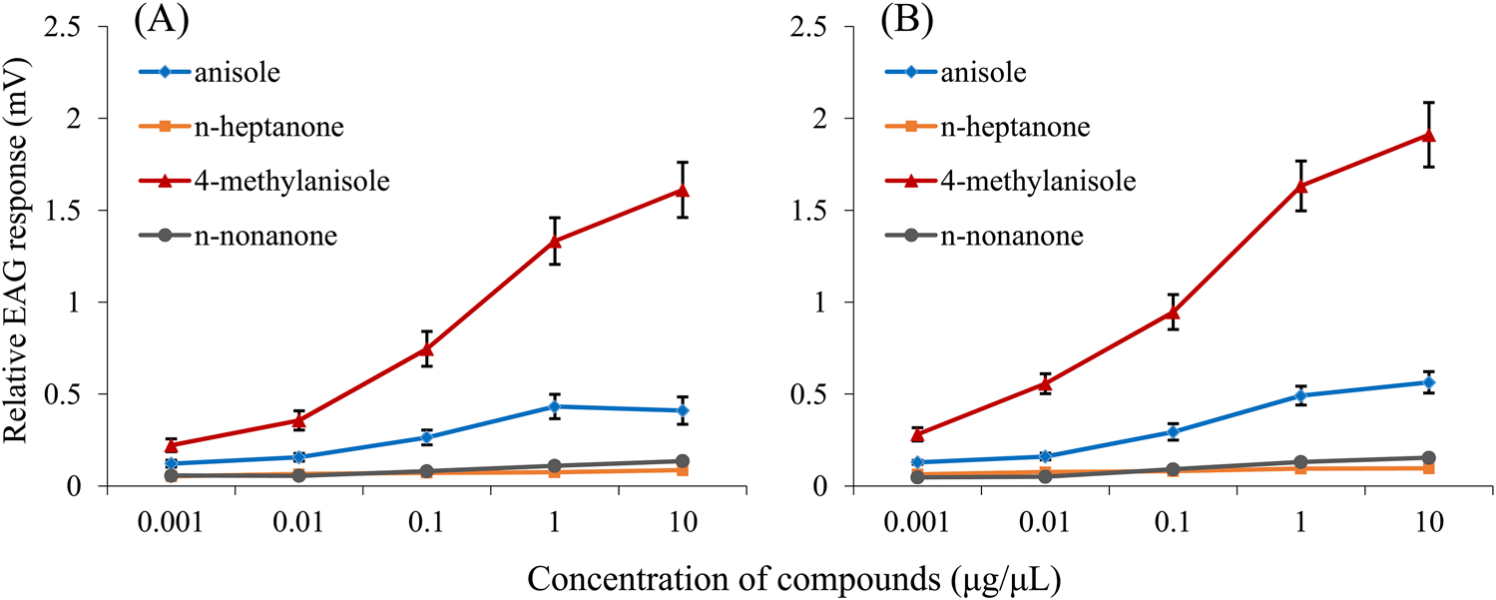
Dose-dependent EAG response curves to four volatile components. (**A**) male antennae; (**B**) female antennae.

The EAG tests showed that the antennae of both male and female WSFCs responded strongly to 4-methylanisole, and responded mildly to anisole (Fig. 3). The EAG responses to 4-methylanisole and anisole showed a positive dose–response relationship. However, n-heptanone and n-nonanone elicited no response from the WSFC antennae, even though we added up to 100 μg of pure compounds. Based on the antennal response tests, we preliminarily regarded 4-methylanisole and anisole as candidate aggregation pheromones of WSFCs.

### Y-tube behavior tests

**Figure 4:**
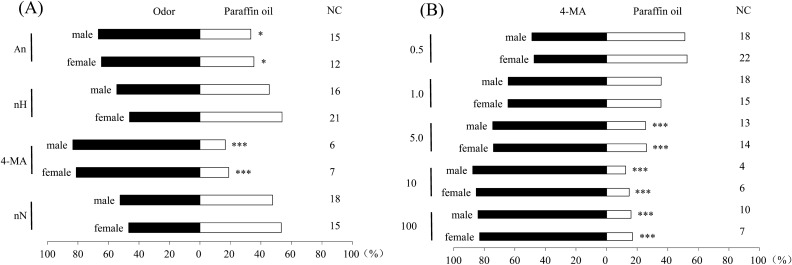
Behavioral responses of *Protaetia brevitarsis* in a Y-tube olfactometer (**A**) to anisole (An), n-heptanone (nH), 4-methylanisole (4-MA) and n-nonanone (nN) at a dose of 10 μg in 10 μL paraffin oil and (**B**) to 4-methylanisole (0.5, 1.0, 5.0, 10, 100 μg in 10 μL paraffin oil). N = 60 for each treatment. NC stands for numbers of non-response beetles. (Binomial tests: * 0.001 < *P* < 0.05; *** *P* ≤ 0.001).

In the Y-tube bioassay, 4-methylanisole was the most attractive candidate at a concentration of 1.0 μg·μL^-1^ (Fig. 4A, male: *χ*^2^ = 24.44, *df* = 1, *P* < 0.001, *N* = 54; female: *χ*^2^ = 21.63, *df* = 1, *P* < 0.001, *N* = 53), followed by anisole (Fig. 4A, male: *χ*^2^ = 5.95, *df* = 1, *P* = 0.015, *N* = 42; female: *χ*^2^ = 4.60, *df* = 1, *P* = 0.032, *N* = 45). Neither sex showed a significant preference for n-heptanone or n-nonanone. Further dose-dependent responses to serial dilutions of 4-methylanisole showed that higher doses of 4-methylanisole attracted more WSFCs of both sexes, and the greatest attraction was observed when > 10.0 μg of pure 4-methylanisole was loaded in paraffin oil (Fig. 4B, male: *χ*^2^ = 32.19, *df* = 1, *P* < 0.001, *N* = 56; female: *χ*^2^ = 27.92, *df* = 1, *P* < 0.001, *N* = 54).

### Aggregation index

**Figure 5:**
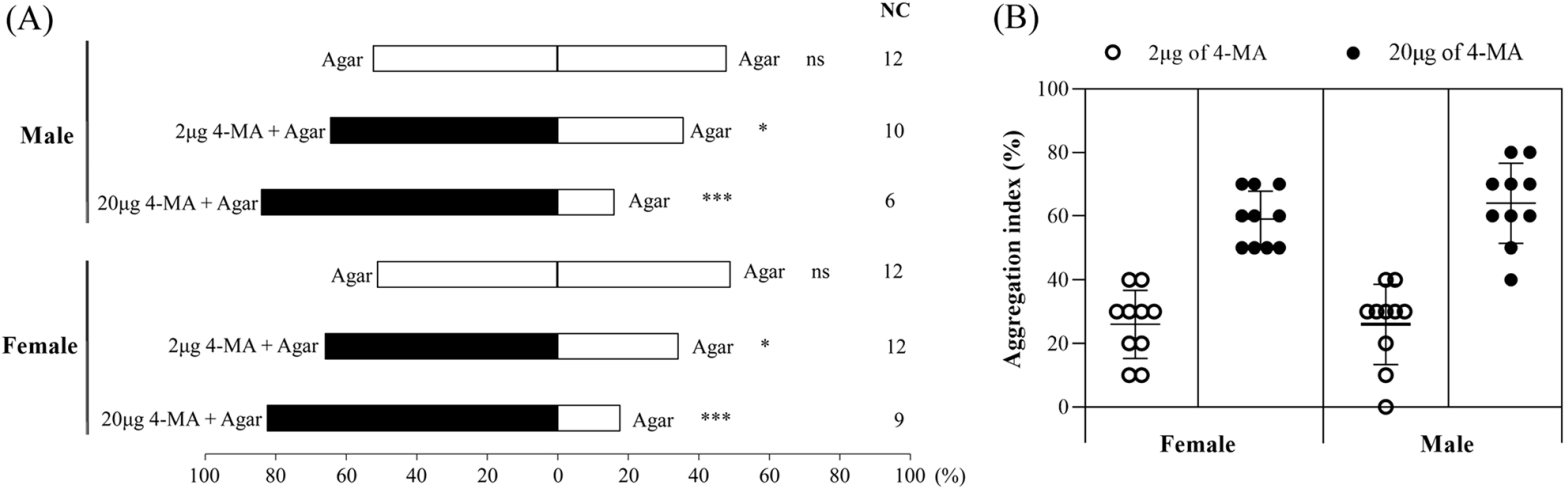
The behavioural preference of *Protaetia brevitarsis* in a dual-choice arena to agar-gel with or without 4-methylanisole (4-MA). (**A**) choice percentage of *P. brevitarsis* to control or treated agar-gel. NC stands for numbers of non-response beetles. (Binomial tests: ns, *P* > 0.05; * 0.001 < *P* < 0.05; *** *P* ≤ 0.001). (**B**) aggregation index of *P. brevitarsis* to 2 μg and 20 μg 4-MA treated agar-gel. The brackets represent standard deviations.

In the two-choice arena without food, the beetles of both sexes flew and crawled around the arena continually, resulting in no significant preference to 4-methylanisole (unpublished data). Inspired from the phenomenon that WSFC aggregations are always observed on their food^1,17,20^, we deduced that WSFCs release aggregation pheromone when they find suitable food sources. In each compartment of the arena, we added odorless agar-gel as food or residence place for WSFCs (Fig. S2). No significant preference between agar-gel only controls (Fig. 5A, male, *χ*^2^ = 0.182, *df* = 1, *P* = 0.670, *N* = 88; female: *χ*^2^ = 0.045, *df* = 1, *P* = 0.831, *N* = 88) indicated that odorless agar-gel was not attractive to either sex of beetles. The majority of WSFCs ate on the 20 μg 4-methylanisole treated agar-gel or stayed in the treated compartment (Fig. 5A, male, *χ*^2^ = 43.574, *df* = 1, *P* < 0.001, *N* = 94; female: *χ*^2^ = 38.253, *df* = 1, *P* < 0.001, *N* = 91), suggesting 4-methylanisole significantly attracted both sexes. The aggregation indexes of 20 μg 4-methylanisole treatment were calculated as 59.00 ± 8.77% (female) and 64.00 ± 12.65% (male), and those of lower doses treatment were 26.00 ± 10.75% and 26.00 ± 12.65% for female and male (Fig. 5B).

### Field trials

To screen the aggregation pheromone of WSFCs, we evaluated the attractiveness of the EAG-active candidates as well as their blends with EAG-inactive components in the field. As the most EAG-active compound, 4-methylanisole, both separately and contained in binary blends, captured > 4 female and > 4 male WSFCs, while blank control, sole n-heptanone and n-nonanone displayed no attractiveness to WSFCs (Table S1). The ratio of captured male to female beetles was near 1:1 (Table S1). The addition of anisole, n-heptanone and n-nonanone to 4-methylanisole failed to improve its attractiveness (male captures: *F*_*3,16*_ = 0.307, *P* = 0.820; female captures: *F*_*3,16*_ = 0.114, *P* = 0.950). We concluded that 4-methylanisole was the sole candidate aggregation pheromone of WSFC.

In the field experiment of dose optimization, loading higher amounts of 4-methylanisole, from 0.5 to 20 mg, significantly increased the WSFC captures (male capture: *F*_*4,20*_ = 17.716, *P* < 0.001; female capture: *F*_*4,20*_ = 17.125, *P* < 0.001. Table S2). The maximum WSFC captures were 11.25 beetles per week at a 4-methylanisole dose of 100 mg, and more 4-methylanisole (> 20 mg) did not contribute significantly to increasing the catch numbers (male captures: *F*_*2,12*_ = 0.995, *P* = 0.398; female captures: *F*_*2,12*_ = 0.588, *P* = 0.571).

### Lure duration

**Figure 6:**
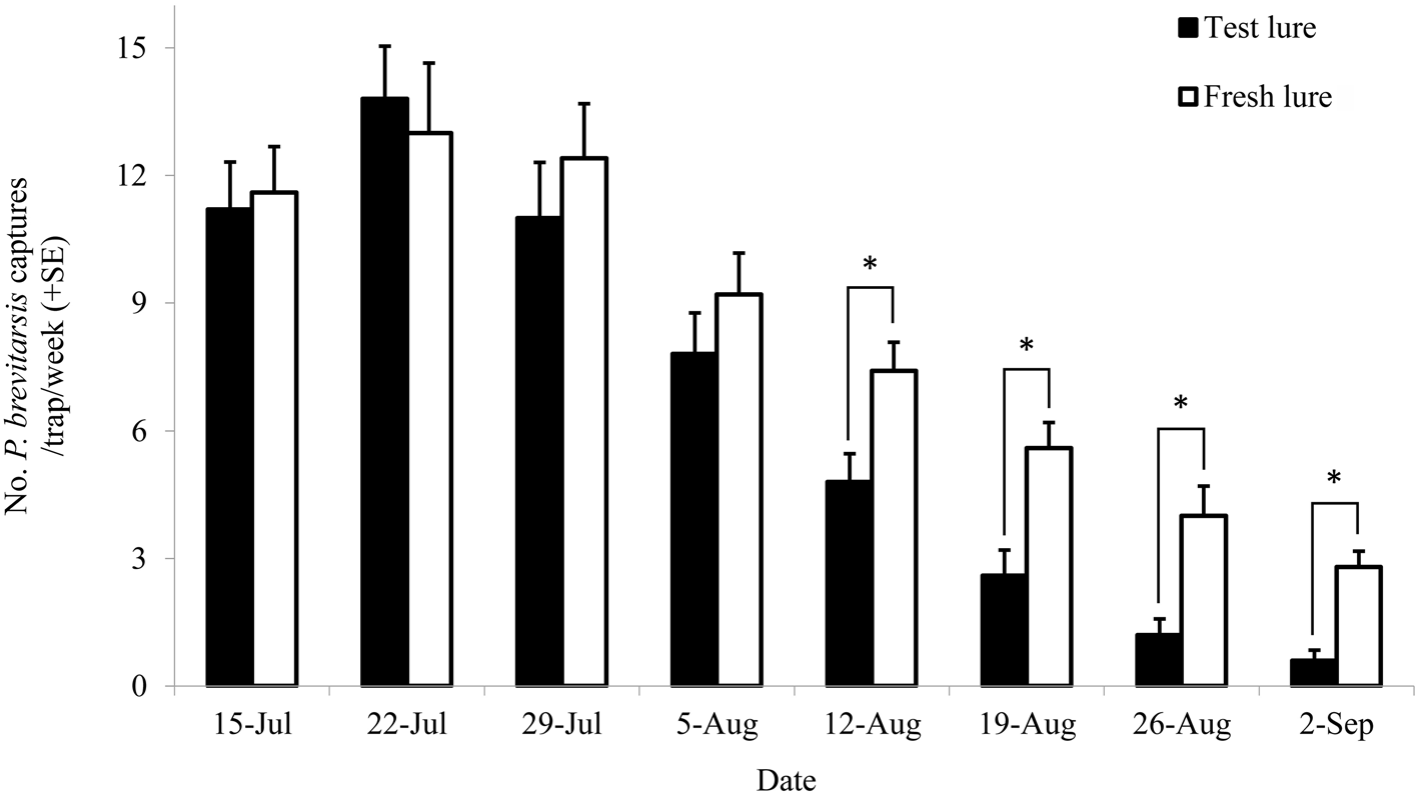
Captures of *Protaetia brevitarsis* adults in traps baited with test lures (with the lures aging during the assays) and fresh lures (changed at a 1-week interval) (mean + SE, N = 5) from 15 July to 2 September, 2018. The asterisks on the bars indicate significant differences in the total catches of female and male WSFCs (*P* < 0.05).

We further evaluated the long-term performance of 4-methylanisole lure from 15 July to 2 September, 2018. There were large differences in the weekly captures of both the fresh lures and test lures, presumably due to the seasonal variation in the WSFC population (Fig. 6). Compared with the fresh lures, however, the test lures trapped almost the same numbers of WSFCs (sum of the captured females and males) in the first four weeks (Fig. 6, 4th week, *F*_*1,8*_ = 1.043, *P* = 0.337). Starting from the 5th week, the test lures attracted significantly lower numbers of WSFCs than the fresh lures (Fig. 6, 5th week, *F*_*1,8*_ = 7.511, *P* = 0.025). Nevertheless, the test lures still trapped approximately three beetles even after aging for 6 weeks, suggesting that the vial lures baited with 4-methylanisole could be an efficient method for long-term monitoring WSFCs.

## Discussion

In the present study, we identified and verified 4-methylanisole as an aggregation pheromone emitted from both sexes of WSFCs. Although producing and emitting aggregation pheromones by both sexes is unusual in beetle species, this phenomenon is reported in *Cydia pomonella* and *Locusta migratoria* and *Colletes* bees^21–23^. Synthetic 4-methylanisole elicited a strong response from adult antennae in the electrophysiology tests, and attracted female and male beetles both in the Y-tube olfactometers and in the field experiments. To our knowledge, 4-methylanisole has not been reported as an aggregation pheromone of any insect.

Although 4-methylanisole is uncommon as an insect-originated volatile, it has been reported in bouquets of many plant species, such as *Melodorum fruticosum*^24^, *Mimusops elengi*^25^, and *Gelsemium sempervirens*^26^. In some species, 4-methylanisole plays crucial roles in the plant-pollinator relationship. As the dominant aromatic volatile in the flowers of *Ficus semicordata* and *Hydrocleys martii*, 4-methylanisole has been proven to contribute to the attraction of pollinators^27,28^. 4-Methylanisole has also been reported in the flowers of phytelephantoid palms (*Arecaceae*)^29^ and *Cymbopetalum brasiliense*^30^, and was inferred to be well perceived by various insects, especially pollinators^28^. In the inflorescences of the macauba palm, the dominant floral fragrance was identified as 4-methylanisole (> 97% relative amount), which showed exclusive attractiveness to a florivorous chafer, *Cyclocephala forsteri*^31^. In this study, we found that 4-methylanisole showed significant attractiveness to the WSFCs, a Cetoniinae species. It is well known that Cetoniinae beetles, the most frequent flower visitors, not only play an important role in plant pollination^32–34^ but also are serious pests of flowers and fruits because they usually attack and feed on blooming flowers and ripe fruits^35^. Polyphagous WSFC adults feed on the flowers and fruits of dozens of plant species^1^, resulting in the possibility that 4-methylanisole identified from WSFC volatiles originated in their foods sources. To control for this, we fed the WSFCs in volatile collection with only water. Therefore, the 4-methylanisole detected in the WSFC adults was produced and released autonomously. To investigate where 4-methylanisole secretes, we separated the midguts, hindguts and accessory (colleterial) glands, which were the potential pheromone-producing organs of Scarabeidae beetles^36,37^. Majority of 4-methylanisole and anisole were detected in the accessory glands of both sexes, and they were also detected in hindguts, while only trace of anisole was found in the midguts (Fig. S2).

Our Y-tube bioassays and field experiments suggest that synthetic 4-methylanisole displayed significant attractiveness to both male and female WSFCs. These results could not exclude the possibility that 4-methylanisole is an aggregation-sex pheromone, which also induces attraction of both sexes^38–40^. WSFCs displayed no obvious courtship behavior (e.g. mounting) towards 4-methylanisole in both Y-tube and arena bioassay, indicating that 4-methylanisol was an aggregation pheromone of WSFCs rather than an aggregation-sex pheromone. WSFCs are active in daytime, flying or crawling around the arena unless they are feeding or resting. To settle WSFCs in the behavioral arena, we used odorless agar-gel as food and resident place. The results of aggregation index (Fig. 5) were fully according with the Y-tube tests (Fig. 4) and field trapping (Table S1-S2), collectively confirming that 4-methylanisole was significantly attractive to both sexes of WSFCs. Combined with food, aggregation pheromones also show higher attractiveness in other chafers^16^. It makes sense that the beetles release chemical signals to summon conspecifics during eating, which could provide conspecific individuals with more opportunities to find food sources and mate partners. It provided future possibility to develop more efficient WSFC attractants by the combination of pheromones and host volatiles.

Few reports have documented 4-methylanisole as an insect-derived sex pheromone or aggregation pheromone, but anisole, the homologue of 4-methylanisole, has been identified as the main component of the sex pheromone of the flower chafers *Holotrichia reynaudi*^3,41^ and *H. consanguinea*^42^. In particular, anisole was used to capture a large number of female and male *H. consanguinea*, leading to aggregation and copulation behavior^6,42^. Anisole was also detected in the volatiles emitted from WSFC adults, but it elicited only a relatively small antennal response (Fig. 3A) and did not result in the enhancement of the attractiveness of 4-methylanisole (Table S1). Whether anisole has biological function will be further assayed in our future study.

It is well documented that flower-derived 4-methylanisole attracts flower-visiting insects, including various kinds of bees and beetles^28,29,31^. This means that traps baited with 4-methylanisole could capture pollinators in the field. During our experiment in a vineyard, a variety of nontarget insects, such as wasps, chafers, ladybirds, spiders, flies, lacewings, moths, hoverflies, leafhoppers and plant bugs, were caught. Compared with the traps baited with sunflower oil only, the traps with 4-methylanisole trapped significantly more halictid bees, *Xylocopa appendiculata* and *Oxycetonia jucunda* Faldermann (unpublished data), which are well-known pollen consumers^43–45^. The capture numbers of other nontarget species were not different between the traps baited with 4-methylanisole and the control lure (unpublished data), suggesting that the capture of these bycatches was not associated with WSFC pheromone.

The capture of beneficial insects would potentially decrease the application of WSFC lures. Novel traps might be a way to reduce the negative effects of control measures on nontarget species. To monitor and control Scarabaeidae pests, many traps, such as cone trap cross-vane panel trap^46^, pitfall trap^47^, funnel trap^48,49^ and Trece trap^50,51^, have been designed and applied. Few studies have reported the use of sticky traps for flower chafers, presumably because chafers are strong enough to escape from sticky cards, a behavior that has been observed in WSFCs. Additionally, color of traps is a significant factor on bycatch rate of nontarget beneficial insects, including honey bees, bumblebees, solitary bees and ladybirds^52,53^. In our further work, we will design and develop more efficient traps with non-attraction for beneficial insects.

Our results indicate that 4-methylanisole, the exclusive field-active component among the volatiles released from the WSFCs, is the sole aggregation pheromone. The vial lures loaded with 4-methylanisole significantly trapped both sexes of WSFCs, suggesting they could be developed as potential method for monitoring this pest. Our future studies will focus on modifying the structure of 4-methylanisole to increase its selectivity in favor of target over non-target insects.

## Methods and materials

### Insects and sample collection

The WSFC population was reared at Cangzhou Technical College (Hebei Province, China). The pupae were separated and individually kept in glass vials until adult emergence. The newly-emerged adults were sorted by sex based upon differences in morphology of their abdomens and reared individually in small cages. Both the male and female adults were given sucrose water (5%) as food after their emergence. WSFCs used for collecting volatiles were fed with only water for three days before volatile collection, to ensure no food odor in the tested beetles. The beetles used in the EAG test and behavioral experiment were fed fresh apple slices, which were replaced every day. The male and female WSFCs were reared in different rooms to avoid an effect of interaction on pheromone collection. The rearing room was maintained at 25 ± 2 °C and 50–70% RH with a 12:12 h L:D.

The head space method^54^ was used to collect the volatiles emitted from virgin WSFCs. Briefly, groups of 50 males and females (~ 10 days old, at the onset of showing aggregation habit^23^) were placed separately in glass aeration chambers (30 cm height × 5.5 cm diam) after rinse in distilled water and natural air-dry for 1 h. The volatiles from the caged beetles were drawn through the chamber using a diaphragm pump at a flow rate of 200 mL·min^−1^, and adsorbed on a purified absorbent (Porapak Q, Sigma-Aldrich, USA). Subsequently, the adsorbed volatiles were eluted with distilled hexane. Air through an empty chamber was also collected as a blank control. The samples were stored at − 20 °C until GC–MS analysis. The collection of volatiles was conducted during the photo period (from 4 to 8 h), considering that the WSFCs naturally feed and mate in the daytime^55^. The volatiles from each sex were collected in triplicate.

### Gas chromatography–mass spectrometry analysis

The collected volatile samples were analyzed on a GC–MS (7890A-5975C, Agilent Technologies, USA) equipped with a DB-5 column (30 m × 0.25 mm × 0.25 μm, Agilent). Helium at a rate of 1.0 mL·min^−1^ was used as the carrier gas. The oven was held at 40 °C for 1 min, set to 120 °C (5 °C·min^−1^) and held for 2 min, then set to 250 °C (10 °C·min^−1^) and held for 5 min. The temperature was held at 250 °C for the injector and 250 °C for the GC–MS interface. The operating conditions for the MS were as follows: ion source temperature, 230 °C; ionization current, 100 μA; ionization energy, 70 eV; accelerating voltage, 6 kV; and scan range, 30–350 *m/z*. The mass spectra were compared to those in the NIST14 database. The identification of candidate aggregation pheromones was confirmed by a comparison of the retention times and mass spectra of authentic standards.

### Chemicals

Anisole (99.5% purity), 4-methylanisole (99% purity), 2-heptanone (98% purity), and 2-nonanone (99% purity) were purchased from Aladdin Chemical Co., Ltd. (Shanghai, China).

### EAG bioassays

The electrophysiological responses of WSFC antennae to the four volatiles from the beetles were monitored using an EAG apparatus (Syntech Ltd., Kirchzarten, Germany). The EAG preparation followed that used in Zhang et al.^56^. In brief, completely amputated antennae of WSFC adults (~ 10 days-old), with the excision of each tip were affixed to the recording electrode with electrically conductive gel. Then, a humidified airstream (~ 200 mL·min^−1^) carried volatiles from serial dilutions (0.01, 0.1, 1.0, 10 and 100 μg of pure compound in 10 μL of paraffin oil) flowing over the antennae at 30 s intervals controlled by an air stimulus controller (CS-55). Paraffin oil was used as a negative control. Each compound was tested on five antennae with five stimuli for each antenna.

### Behavioral experiment

The responses of the beetles to each volatile were tested in a Y-tube olfactometer with a diameter of 3 cm, 25-cm long stems and 20-cm long arms (at an angle of 60°). Humidified and purified air was pumped into the olfactometer by an atmosphere sampling instrument (QC-1B, 0–500 mL·min^−1^, Beijing Municipal Institute of Labor Protection, Beijing, China) at 100 mL·min^−1^. The odor compounds were dissolved in paraffin oil. Paraffin oil was used as a negative control. In the Y-tube olfactometer, compound solution and paraffin oil on strips of filter paper (3 cm × 0.5 cm) were placed into the arms, and a WSFC individual (10–20 days old) was released into the stem during the photophase (WSFCs are diurnal insects^23^). The orientation of the beetle was recorded after 15 min at 25–27 °C. The beetles over midpoint of control or odor arms were defined as making choice, and others were recorded as no choice. To eliminate positional bias, the odor arms were rotated after five trials. The Y-tube was washed with ethanol after each test. In each treatment, 60 beetles were introduced into Y-tube individually. The attractive compounds were screened by loading 10 μL of the beetle-produced compounds at a concentration of 1.0 μg·μL^−1^ as odor sources. Then, the dose-dependent response to the candidate aggregation pheromone was evaluated by loading 0.5, 1.0, 5.0, 10 and 100 μg of pure compound in 10 μL of paraffin oil.

### Aggregation index

**Figure 7:**
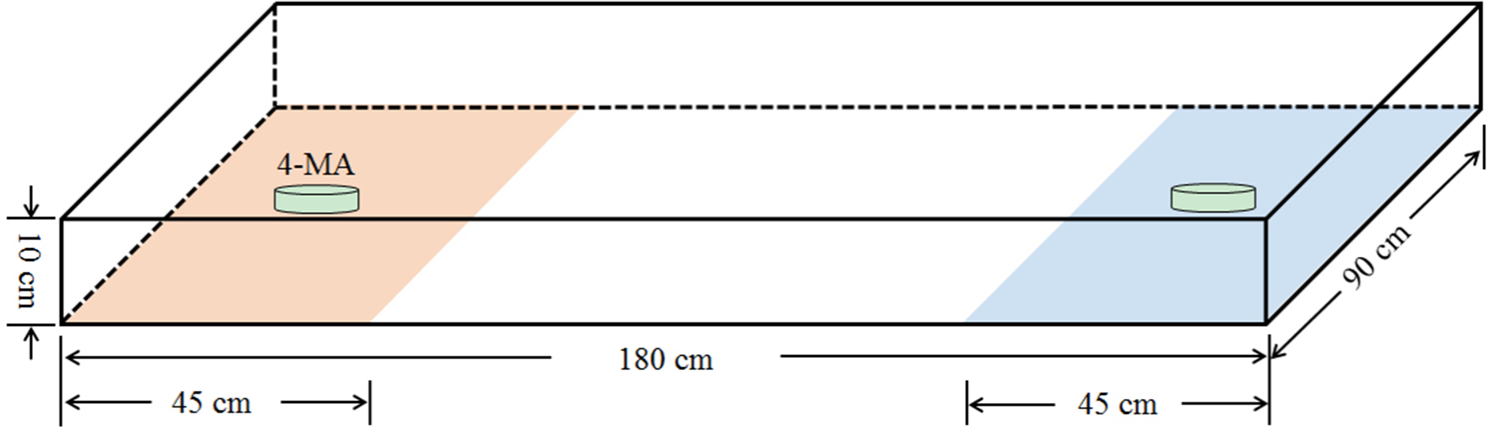
Setup for behavioural preference of *Protaetia brevitarsis* in a dual-choice arena system. The yellow and blue part represented treated and control compartment, respectively. The beetles were initially positioned in the center of the arena. Two short cylinders represent the 9-cm petri dish which is filled with 1.5% agar-gel.

To determine the aggregation index of WSFCs to the aggregation pheromones, we conducted bioassays in an arena of two-choice olfactometer (180 cm × 90 cm × 10 cm) with a gauze to prevent escape (Fig. 7). In the center of treated compartment, a 9-cm petri dish with agar-gel (1.5%) and 2 μg or 20 μg of pure 4-MA was added, while a petri dish with agar-gel was set in the control compartment. The WSFCs in a group of ten were introduced in the center of the arena, and their numbers in each section were counted after 15 min. Uncommitted beetles in the middle arena were recorded as non-responders. Each treatment was replicated 10 times (a total of 100 WSFCs). The aggregation index was calculated as (T − C) × 100/N, where T and C are the numbers of beetles stayed in the treated and control compartments respectively, and N represents the total number of tested WSFCs^57^.

### Lures

Pheromone dispensers were prepared using polyethylene (PE) vials (25 mm × 6 mm × 0.25 mm thick; Pherobio Technology Co. Ltd., Beijing) following previous procedure with slight modifications^58^. Briefly, the PE vials were loaded with appropriate amounts of the candidate aggregation pheromone components in 200 μL of sunflower oil and heat-sealed. The volatiles slowly permeated the vial walls and were released.

### Traps

In all field experiments, bucket traps (Pherobio Technology Co. Ltd., Beijing) were employed to capture WSFCs. The traps consisted of a yellow canopy with a green lure holder, a yellow infundibulate section, and a transparent gather bucket for captured insects^58^ (Fig. S3). Unlike our previous application for plant bugs, no water was added to the collection buckets.

### Field trials

Field tests were conducted in a 10-ha vineyard in Baoding (Hebei Province, China, 38.58°N; 115.49°E). Traps were hung ~ 1.5 m above the ground between the rows of vines with a minimum of 20 m apart. In each experiment, traps were randomly arranged in the vineyard. Traps were examined weekly, and the numbers of captured beetles were counted and recorded by sex. After removing the trapped WSFCs and renewing the lures, the positions of the traps were rerandomized. Five replicates were tested for each treatment.

### Verification of WSFC pheromones

Live beetles were used to verify the existence of long-distance pheromones in WSFCs from 26 July to 9 August 2016. Five live WSFCs (~ 10 days old) were marked with red markers colors and caged directly in every trap. Fresh apple slices were also added as food for the WSFCs. Six treatments were conducted: blank, food only, virgin females, virgin females, mated females and mated males. Every morning, the live beetles and their food were renewed, and the traps washed.

### Attractiveness evaluation of candidate pheromone

The attractive activities of volatile compounds emitted from WSFCs were determined in the same vineyard from 18 July to 17 August 2017. Eight treatments were designed including anisole, 4-methylanisole, 2-heptanone, 2-nonanone, 4-methylanisole + 2-heptanone, 4-methylanisole + anisole, and 4-methylanisole + 2-nonanone, and sunflower oil was used as negative control. The dose of each component was 20 mg per lure.

### Dose optimization of aggregation pheromone

The dose-dependency of the attractiveness of the WSFC aggregation pheromone were assessed by loading vials with serial doses of 4-methylanisole (0.5, 1, 5, 10, 20, 50 and 100 mg) from 11 August to 10 September 2017. Vials loaded with sunflower oil were used as negative controls.

### Long-term assessment of lures

The longevity of the vial lures were estimated by continuously testing the attractiveness of the lures from 15 July to 2 September 2018. All the vial lures were prepared by loading 100 mg of 4-methylanisole. Due to the lack of efficient measures to monitor the WSFC population, a treatment baited with fresh lures (FLs) that were renewed after each check was set to avoid the deviation of WSFC captures originating from the population variation.

### Statistical analysis

SPSS 19.0 was used for all the statistical analyses. The Y-tube and binary-choice arena data were analyzed using χ^2^ goodness-of-fit tests after excluding the unresponsive individuals. The differences in captures between pairs of treatment were analyzed by one-way analysis of variance (ANOVA) followed by Tukey’s test. *P* < 0.05 was considered statistically significant. The data used for ANOVA did not include the all-zero values from controls and failed treatments, and were validated with Levene’ test.

## Supplementary Information

Supplementary Information.
