# Supplementary material for: Identification and field verification of an aggregation pheromone from the white-spotted flower chafer, Protaetia brevitarsis Lewis (Coleoptera: Scarabaeidae)
Source: Sci Rep. 2021 Nov 16;11:22362. doi: 10.1038/s41598-021-01887-y (PMC8595700; doi:10.1038/s41598-021-01887-y)
Supplement: Supplementary file 1 — Supplementary Information. [file 41598_2021_1887_MOESM1_ESM.docx]

Identification and field verification of an aggregation pheromone from the white-spotted flower chafer, *Protaetia brevitarsis* Lewis (Coleoptera: Scarabaeidae)

Xiaofang Zhang^1^, Liuyang Wang^2^, Chunqin Liu^3^, Yongqiang Liu^2^, Xiangdong Mei^2^, Zhongyue Wang^2^, Tao Zhang^1,^*

**^1^** Institute of Plant Protection, Hebei Academy of Agriculture and Forestry Sciences, Integrated Pest Management Center of Hebei Province, Key Laboratory of IPM on Crops in Northern Region of North China, Ministry of Agriculture, Baoding 071000, China

**^2^** State Key Laboratory for Biology of Plant Diseases and Insect Pests, Institute of Plant Protection, Chinese Academy of Agricultural Sciences, Beijing 100193, China

**^3^** Cangzhou Technical College, Cangzhou, 061001, P. R. China

* **Corresponding to:** cauzht@163.com


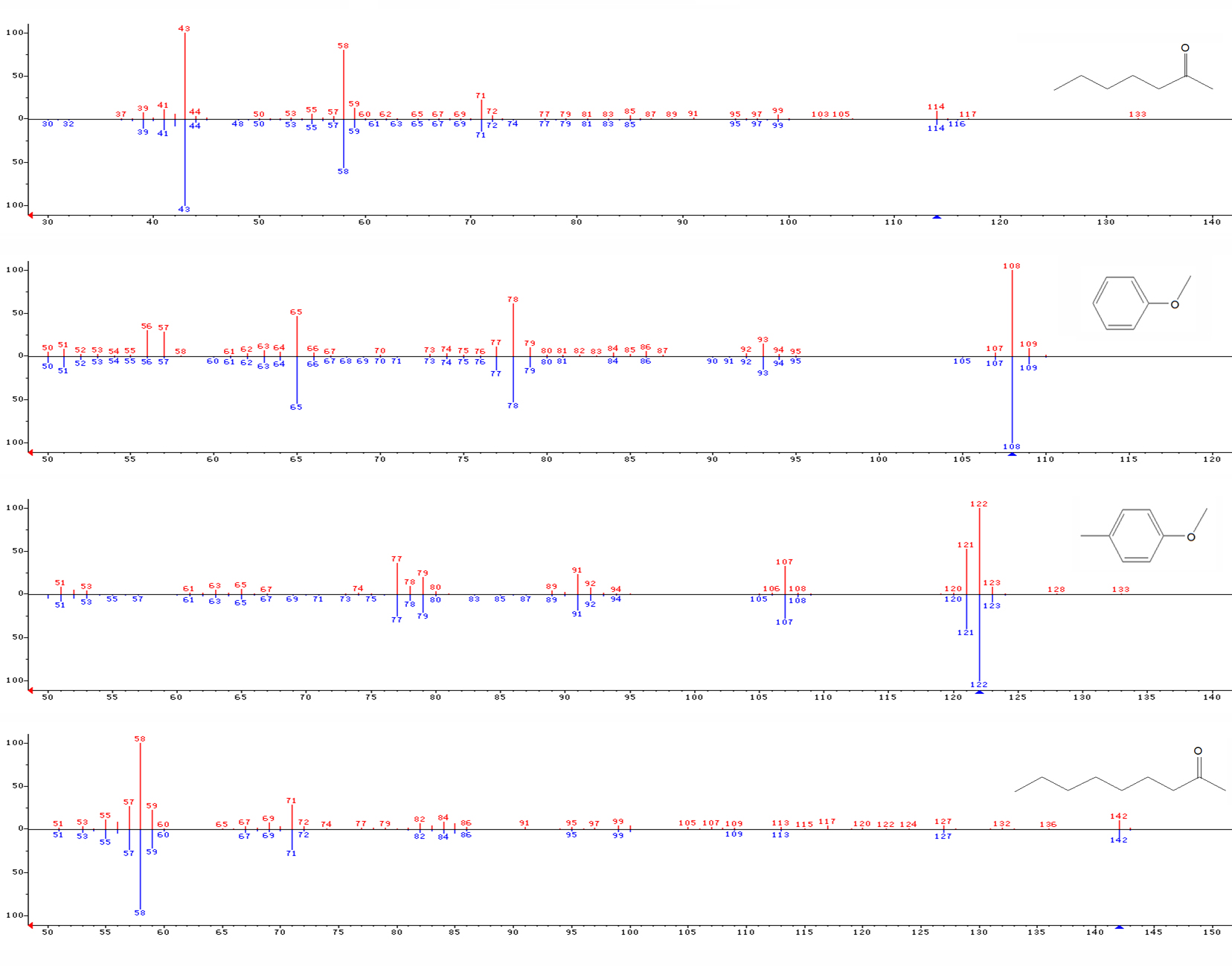


Figure S1 Mass spectra of n-heptanone, anisole, 4-methylanisole and n-nonanone from volatiles of *Protaetia brevitarsis* (Red), in comparison of authentic compounds (Blue).


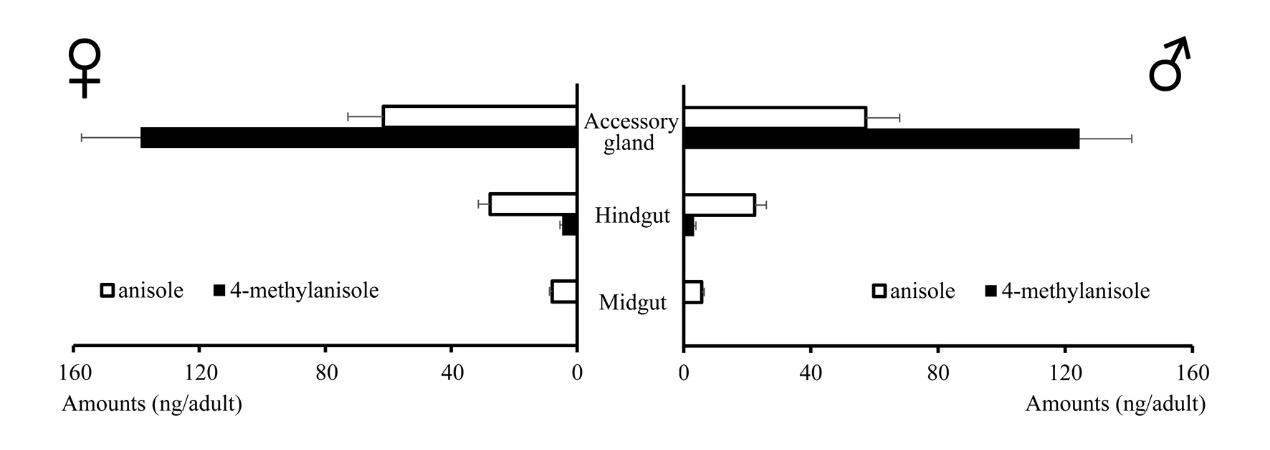


Figure S2 Amounts of anisole and 4-methylanisole from accessory glands, hindguts and midguts of *Protaetia brevitarsis.*

Figure S3 Bucket traps used in field trapping. Green section at the center of canopy is the lure holder. Beetles attracted by lures slide into the transparent gather bucket through the yellow infundibulate section.

**Table S1** Captures of *Protaetia brevitarsis* in traps baited with synthetic compounds identified from adult volatiles from 18 July to 17 August, 2017

| Treatments | No. of Males  (Mean±SE, *N* = 5) | Statistics* | No. of Females  (Mean±SE, *N* = 5) | Statistics* |
| --- | --- | --- | --- | --- |
| control | 0 | DF = 4,20  *F* = 11.123  *P* < 0.001 | 0 | DF = 4,20  *F* = 9.599  *P* < 0.001 |
| 4-methylanisole | 4.25±0.73a |  | 4.35±0.70a |  |
| anisole | 0.10±0.06b |  | 0.20±0.09b |  |
| n-heptanone | 0 |  | 0 |  |
| n-nonanone | 0 |  | 0 |  |
| 4-methylanisole + anisole | 4.40±0.56a |  | 4.55±0.75a |  |
| 4-methylanisole + n-heptanone | 4.35±0.59a |  | 4.60±0.66a |  |
| 4-methylanisole + n-nonanone | 4.20±0.62a |  | 4.25±0.62a |  |

The same letters in a column indicate no significant differences in the number of WSFCs captured (*P* < 0.05).

* The all-zero values from controls and failed treatments were not included in the ANOVA.

**Table S2** Captures of *Protaetia brevitarsis* in traps baited with serial doses of 4-methylanisole from 11 August to 10 September 2017

| Treatments | No. of Males  (Mean±SE, *N* = 5) | Statistics* | No. of Females  (Mean±SE, *N* = 5) | Statistics* |
| --- | --- | --- | --- | --- |
| control | 0 | DF = 6,28  *F* = 13.917  *P* < 0.001 | 0 | DF = 6,28  *F* = 13.411  *P* < 0.001 |
| 0.5 mg | 0.85±0.26de |  | 0.93±0.31de |  |
| 1 mg | 1.40±0.28cde |  | 1.60±0.36cde |  |
| 5 mg | 2.20±0.46cd |  | 2.25±0.45cd |  |
| 10 mg | 3.10±0.41bc |  | 3.25±0.25bc |  |
| 20 mg | 4.85±0.42ab |  | 5.05±0.52ab |  |
| 50 mg | 5.15±0.71a |  | 5.55±0.74a |  |
| 100mg | 5.45±0.76a |  | 5.80±0.87a |  |

The same letters in a column indicate no significant differences in the number of WSFCs captured (P < 0.05).

* The all-zero values from controls and failed treatments were not included in the ANOVA.
